# Determining the structure of the bacterial voltage-gated sodium channel NaChBac embedded in liposomes by cryo electron tomography and subtomogram averaging

**DOI:** 10.1038/s41598-023-38027-7

**Published:** 2023-07-17

**Authors:** Shih-Ying Scott Chang, Patricia M. Dijkman, Simon A. Wiessing, Misha Kudryashev

**Affiliations:** 1 Max-Delbrück-Center for Molecular Medicine in the Helmholtz Association (MDC), In Situ Structural Biology, Berlin, Germany; 2 Max Planck Institute of Biophysics, Frankfurt on Main, Germany; 3 Buchmann Institute for Molecular Life Sciences, Goethe University of Frankfurt on Main, Frankfurt on Main, Germany; 4 Institute of Medical Physics and Biophysics, Charité-Universitätsmedizin Berlin, Berlin, Germany

**Keywords:** Electron microscopy, Molecular biophysics, Ion channels

## Abstract

Voltage-gated sodium channels shape action potentials that propagate signals along cells. When the membrane potential reaches a certain threshold, the channels open and allow sodium ions to flow through the membrane depolarizing it, followed by the deactivation of the channels. Opening and closing of the channels is important for cellular signalling and regulates various physiological processes in muscles, heart and brain. Mechanistic insights into the voltage-gated channels are difficult to achieve as the proteins are typically extracted from membranes for structural analysis which results in the loss of the transmembrane potential that regulates their activity. Here, we report the structural analysis of a bacterial voltage-gated sodium channel, NaChBac, reconstituted in liposomes under an electrochemical gradient by cryo electron tomography and subtomogram averaging. We show that the small channel, most of the residues of which are embedded in the membrane, can be localized using a genetically fused GFP. GFP can aid the initial alignment to an average resulting in a correct structure, but does not help for the final refinement. At a moderate resolution of ˜16 Å the structure of NaChBac in an unrestricted membrane bilayer is 10% wider than the structure of the purified protein previously solved in nanodiscs, suggesting the potential movement of the peripheral voltage-sensing domains. Our study explores the limits of structural analysis of membrane proteins in membranes.

## Introduction

Biological membranes provide physical barriers in cells and organelles, and allow the maintenance of electrochemical gradients that can be used to trigger various membrane proteins for physiologically critical functions^1,2^. Voltage-gated ion channels (VGICs) are a class of transmembrane proteins that are selectively permeable to ions such as Na^+^, K^+^, Ca^2+^, and Cl^-^ when they are activated and undergo conformational changes due to changes in membrane potential around the channels^3^. In neuronal signaling and muscular contraction, Na^+^ influx through voltage-gated sodium channels (VGSCs) corresponds to the rapid rising phase of the action potential in the membranes of neurons and other electrically excitable cells^4,5^ VGSCs show a series of conformational changes between open, closed, and inactivated states during a cycle of an action potentia^6,7^. Each action potential is followed by a refractory period, during which the VGSCs enter an inactivated state while the Na^+^ and K^+^ ions return to their resting state distributions across the membrane, yielding a resting potential of approximately − 70 mV in most neurons. Under the resting potential, VGSCs transition back to their resting state in which they remain until the next action potential.

NaChBac is a prokaryotic VGSC from *Bacillus halodurans* widely used as a model to study the structure and function of VGSCs. In contrast to the eukaryotic VGSCs which are made of four linked homologous subunits, prokaryotic VGSCs are tetramers formed from four identical individual subunits. Each subunit consists of 6-transmembrane helices; helices S1-S4 form the voltage-sensing domains for each unit, while S5 and S6 of the four subunits together form the common pore domain^8^ X-ray crystallography and recent single particle cryo electron microscopy (cryo-EM) analysis provided mechanistic insights into the activation sequence of VGICs. Crystal structures of the prokaryotic Na_v_Ms from *Magnetococcus marinus* were determined in an open state, with an open selectivity filter leading to an open activation gate at the intracellular membrane surface allowing permeation by hydrated sodium ions^9,10^. The crystal structure of Na_v_Ab from *Arcobacter butzleri* was captured in a closed-pore conformation by amino acid substitutions^11^, and the cryo-EM structure of NaChBac in nanodiscs showed a potentially inactivated state^12^. For eukaryotic VGSCs, cryo-EM structures were obtained of Na_v_1.4 in complex with the β1 subunit from electric eel and human in an open pore state^13,14^, and of Na_v_PaS from American cockroach in multiple closed conformations^15^. In order to stabilize the resting conformation of VGSCs for cryo-EM structure determination, disulfide crosslinking in the voltage-sensing module was used for Na_v_Ab^16^. Also, tarantula toxin and voltage-shifting mutations were designed for trapping the resting state of a Na_v_1.7 chimera^17^. The structures of the potassium channels Eag and KCNQ1 under membrane potential have recently been reported, describing conformational changes in the voltage sensing domain and the interplay between the channel and lipids^18,19^. Despite the great progress made, a structure of a VGSC under a physiological resting membrane potential to provide support for the classical ‘‘sliding helix’’ model for gating from the resting state to the activated state is still missing^16^. Indeed, for small (120 kDa) VGSC structural analysis in unrestricted membranes in presence of the electrochemical gradients is still challenging.

Proteoliposomes, which are lipid vesicles containing reconstituted membrane proteins, provide an excellent system for functional and structural studies of membrane proteins under conditions that mimic those *in vivo*^20^. Proteoliposomes allow preservation of the functional lipid environment, generation of transmembrane ionic gradients and do not restrict the motion of transmembrane helices in the membrane plane. Despite extensive functional characterizations using proteoliposomes, successful attempts to employ this system for structural elucidation of membrane proteins are still limited. Structures of hBK channel^21,22^, AcrB transporter^23^, Eag K_v_ channel^18^, and PIEZO1 channel^24^ in liposomes have been determined by single particle cryo-EM at resolutions ranging from 3.5 to 7 Å. However, without a significantly large soluble domain, it may be challenging to “identify” fully transmembrane proteins in electron micrographs for alignment and averaging^23^. Indeed, the molecular weight of these proteins for which structures were solved from proteoliposomes is relatively high: hBK channel is ~ 500 kDa, AcrB transporter is ˜350 kDa, Eag K_v_ channel is ˜390 kDa, and Piezo1 channel is ˜860 kDa, and they have substantial extramembranous domains.

Cryo electron tomography (cryo-ET) and subtomogram averaging (StA) have also been used for determining structures of membrane proteins in proteoliposomes: structures of the small membrane protein MspA (˜160 kDa) and the serotonin receptor ion channel 5-HT_3_R (˜275 kDa) were determined at resolutions of ˜17 and ˜12 A respectively in 2012 and 2015^25,26^. Later, a structure of a large ion channel RyR1 was reported in situ first at 12.6 Å resolution and then at subnanometer resolution allowing to resolve alpha helices^27,28^. While the resolution of StA structures is lower than typically obtained by the single particle reconstructions, we hypothesized that StA might be able to target smaller membrane proteins in proteoliposomes, as tomograms contain the third dimension compared to 2D imaging in single particle cryo-EM. Here, we attempted to capture the resting state of the small (~ 120 kDa) bacterial VGSC NaChBac by cryo electron tomography. To this end, we purified NaChBac, reconstituted it into liposomes, introduced a transmembrane potential to this system, and performed structural analysis by cryo-ET and subtomogram averaging.

## Results

### Design, sample preparation and reconstitution into proteoliposomes

We produced His_6_-GFP^A206K^-NaChBac in *Escherichia coli* C41(DE3) and purified it using a two-step purification approach of affinity and size-exclusion chromatography (SI Appendix, Fig. S1A–C). GFP was fused to the N-terminal end of each of the channel subunits to function as an "anchor" for identifying the channels in the membrane. We next reconstituted NaChBac into liposomes using *E. coli* polar lipid extract to mimic the native bacterial membrane environment. A relatively low lipid-to-protein ratio of 2:1 (weight to weight) was used in order to maximise the number of particles per proteoliposome and thus the efficiency of data collection. For the reconstitution, the detergent DDM was removed by dialysis, followed by incubation with Bio-beads^29^. We found that reducing the rate of detergent removal improved the reconstitution efficiency, resulting in more protein copies per proteoliposome as observed by cryo-EM. However, due to the very low critical micelle concentration of DDM, a relatively long time for the dialysis (around one week) and subsequent incubation with Bio-beads was necessary, otherwise the resulting proteoliposomes showed the membrane with a “fluid edge” (SI Appendix, Fig. S2B). Gradient ultracentrifugation showed a significant protein band shift for the proteoliposomes compared to empty liposomes, and the 55-kDa his-tagged GFP^A206K^-NaChBac showed no signs of contamination or protein degradation by SDS-PAGE (Fig. 2A).

**Figure 1:**
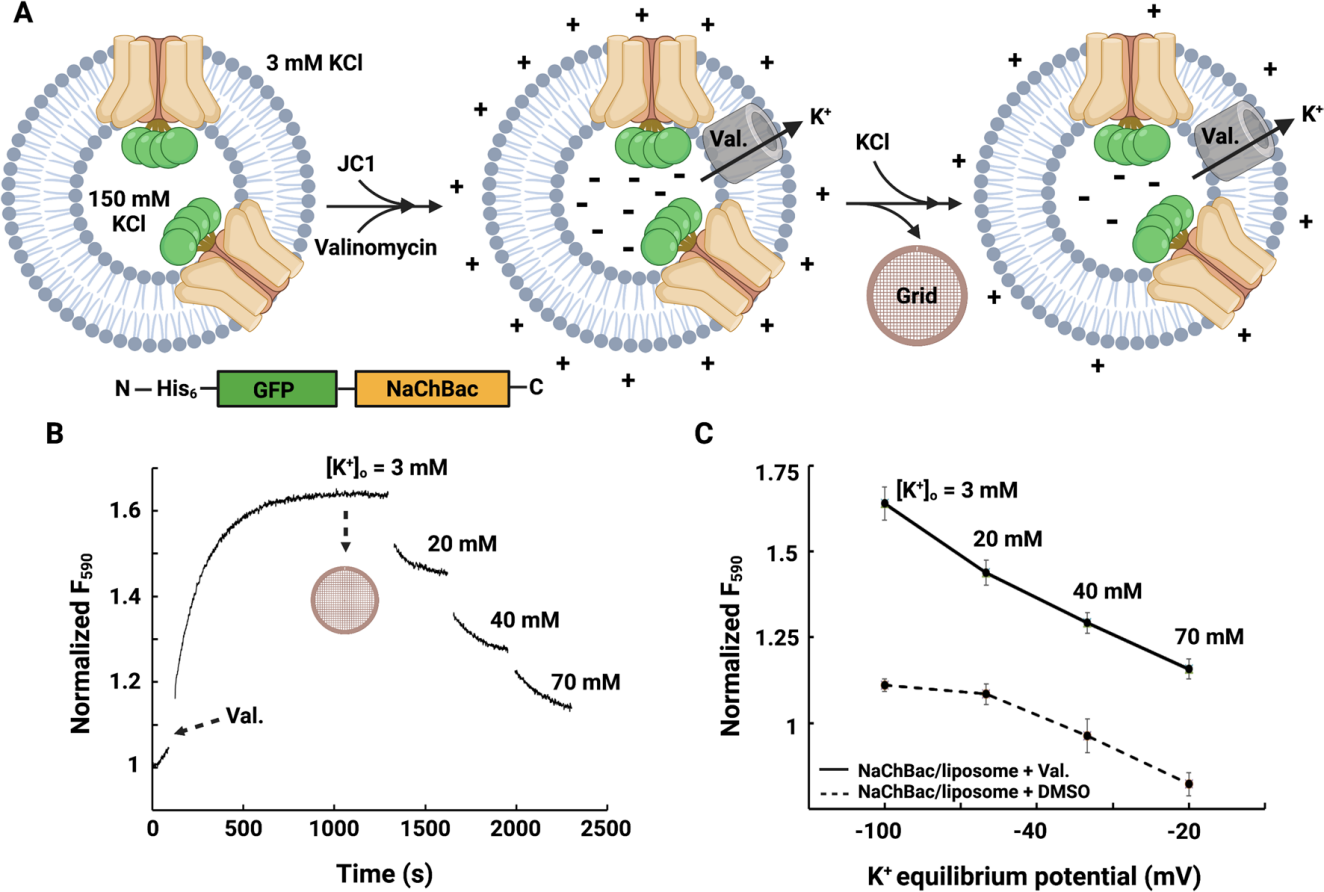
Reconstitution of NaChBac in proteoliposomes and preparation of polarized NaChBac-containing liposomes. (**A**) A schematic of the protocol used to obtain polarized proteoliposomes for cryo-EM analysis. Proteoliposomes loaded with 150 mM KCl were resuspended into 3 mM KCl buffer, and valinomycin was added to mediate potassium flux. Potassium efflux through valinomycin generated a negative potential inside of the proteoliposomes with respect to the outside, and the red fluorescence of JC-1 aggregates was measured. The panels were created with BioRender.com. (**B**) Fluorescence-based liposome flux assay of proteoliposome membrane potential. Addition of valinomycin allowed potassium efflux, and a subsequent decrease in fluorescence as external KCl concentration was increased, indicating K^+^-selective permeability and the stability of proteoliposomes. Proteoliposomes were frozen on gold-supported gold grids for structure determination while [K^+^]_out_ = 3 mM, [K^+^]_in_ = 150 mM, yielding a membrane potential (V_Eq._) of − 100 mV at room temperature (298 K). (**C**) Normalized fluorescence showed the difference between proteoliposomes in the presence of 1 μM valinomycin and DMSO (control), indicating potassium equilibrium potential was triggered by valinomycin. Error bars indicate standard errors (n = 3–5).

In order to generate a membrane potential we used the well-established protocol of resuspending proteoliposomes prepared with a buffer with a high KCl concentration into a buffer with a low KCl concentration followed by addition of a potassium ionophore valinomycin^21^. The membrane potential across the NaChBac-containing proteoliposome bilayer was assayed using the voltage-sensitive cationic fluorescent dye JC-1^30,31^ (Fig. 1A). Proteoliposomes were prepared in a buffer with 150 mM KCl and were resuspended into 3 mM KCl buffer in the presence of 1 μM valinomycin (DMSO as a control experiment), resulting in a membrane potential of − 100 mV at room temperature (298 K) as estimated using the Nernst equation for the potassium diffusion potential calculation^32^. Measuring the fluorescence over time showed stable levels of membrane potential over tens to thousands of seconds. Increasing the external concentration of KCl led to a decrease in JC-1 fluorescence due to a reduced negative membrane potential, indicating the K^+^-selective permeability and the stability of proteoliposomes (Fig. 1B and C). These polarized proteoliposomes, mimicking conditions of the native negative resting membrane potential (− 100 mV), were immediately subjected to structural analysis by (cryo-)EM.

### Cryo-EM/ET and structural analysis by StA

**Figure 2:**
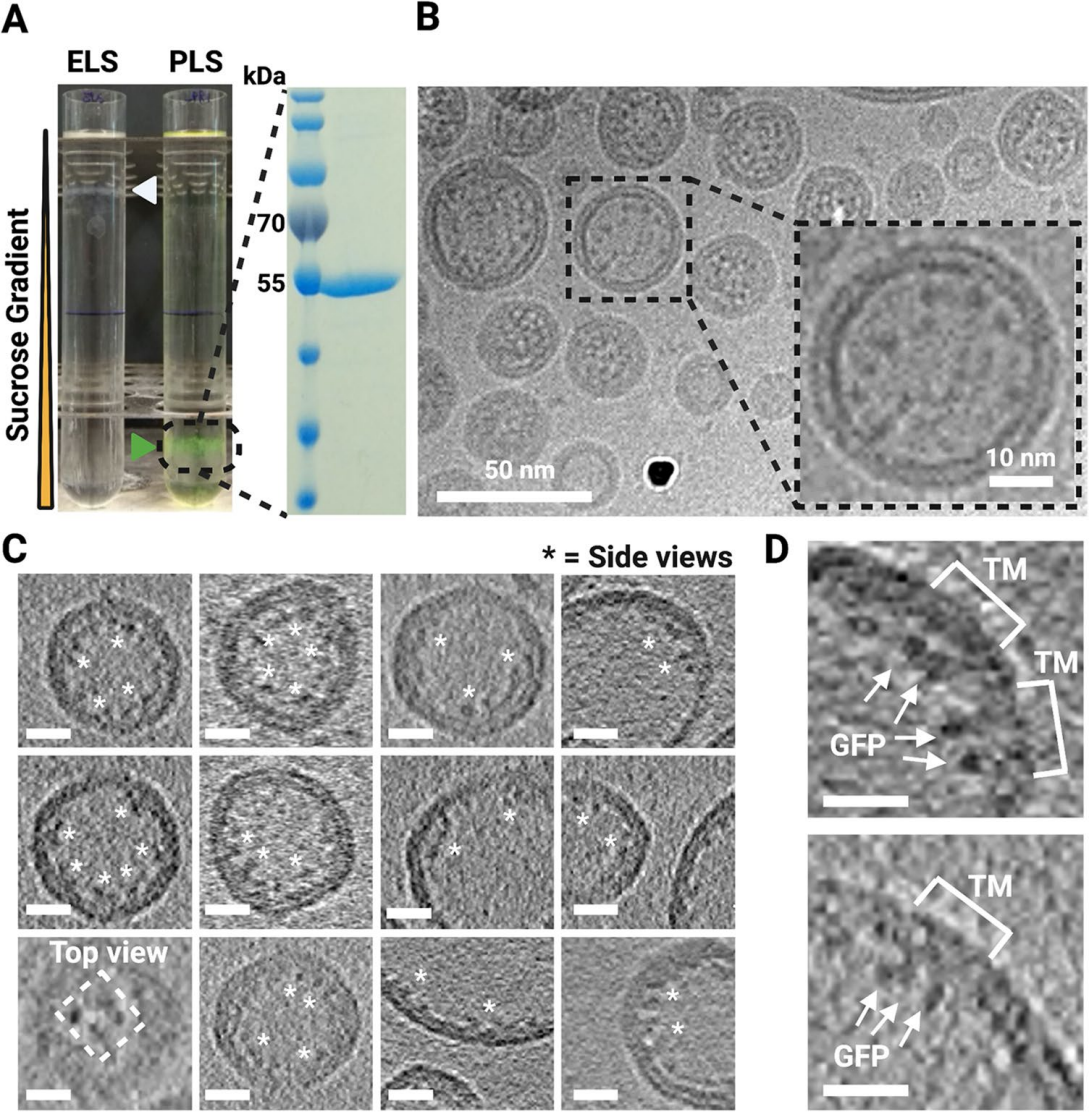
Visualizing NaChBac proteoliposomes by cryo-EM/ET. (**A**) Sucrose gradient ultracentrifugation showed a significant band shift between empty liposomes (ELS) and proteoliposomes (PLS), and the protein showed no degradation on the SDS-PAGE after proteoliposome reconstitution. (**B**) A cryo-image of proteoliposomes. The inset showed a proteoliposome with proteinaceous densities. (**C**) Cryo-ET slices of proteoliposomes showed particle positions and the side view and the top view of his-GFP-NaChBac. White asterisks indicate distinct non-membrane densities. (**D**) Near-equatorial slices through tomograms of proteoliposomes showed GFPs outside the lipid bilayer in the lumen of the proteoliposomes and transmembrane domains of NaChBac embedded in the membrane. Scale bars: 10 nm.

Proteoliposomes were screened by negative stain-EM (SI Appendix, Fig. S2) and cryo-EM showing a large population of proteoliposomes with a diameter of up to 150 nm. While some proteoliposomes were much larger, the smaller proteoliposomes were more amenable to producing thin ice for cryo-ET imaging. The initial cryo-EM preparations showed a significant clustering of proteoliposomes next to the edges of the grid holes with empty hole centers. In order to produce a uniform sample distribution, we performed multi-sample application on gold-supported gold grids inspired by previous reports^33,34^, tuning the parameters of the sample preparation robot, resulting in the final sample for cryo-ET (Fig. 2B).

We collected 98 tomograms (Table S1) and processed them using early versions of tomoBEAR^35^ combining Motioncor2^36^, Gctf^37^, Dynamo^38^ and IMOD^39^. Tomographic reconstructions clearly showed GFP densites and occasional densities between the membranes. The majority of the particles had the GFP "in the lumen of the proteoliposome”.Knowledge of the orientation of the majority of the channels defined how we established the voltage across the membrane, *i.e.* directed similarly to potential found across the inner membrane of the native bacterium. We could observe "side-views" and tetramer-resembling "top-views" with putative GFP densities positioned closely to the membrane (Fig. 2 C and D). Unfortunately due to the small size of NaChBac we could not perform automated particle picking. Attempts to use template matching or geometry-supported particle identification by "drawing" membranes followed by classification of positions into protein-containing and empty membranes were unsuccessful. We therefore picked ˜86,000 tomographic positions from the cryo-ET volumes (prioritizing tomograms with thin ice) manually using the Dynamo catalogue system^40^.

**Figure 3:**
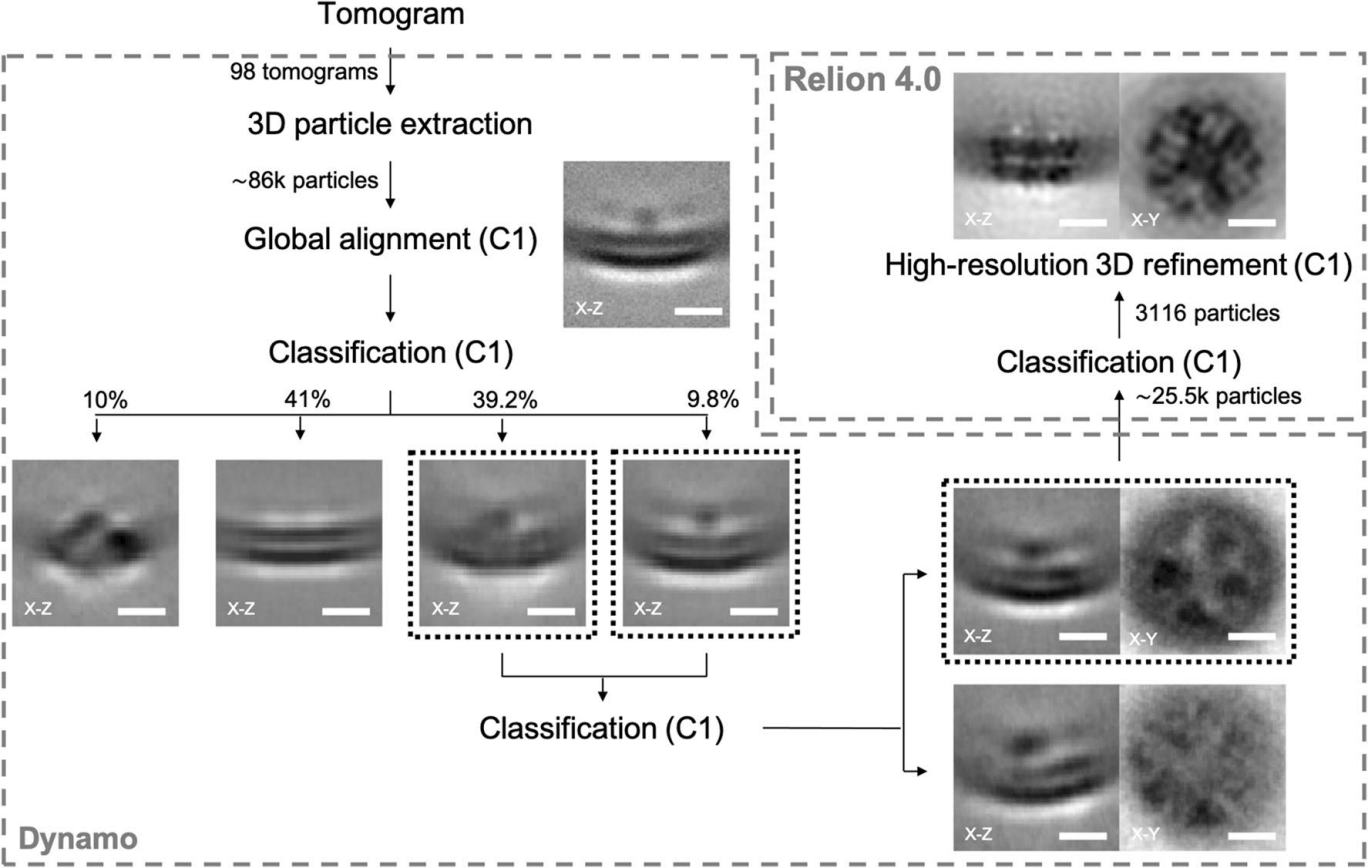
The workflow for structural determination of NaChBac embedded in liposomes by StA. Manually identified particles (˜86,000) were extracted from 98 tomograms. After a global alignment of all extracted particles, multiple rounds of classification were applied to the classes containing tetrameric features from the top-views. Particles with clear protein signals were then subjected to the RELION-4.0 for the next classification and refinement. Final subtomogram averaging from 3116 particles displayed clear domain features of NaChBac. (Scale bars: 5 nm.) More details were provided in SI Appendix, Fig. S3 and S4.

**Figure 4:**
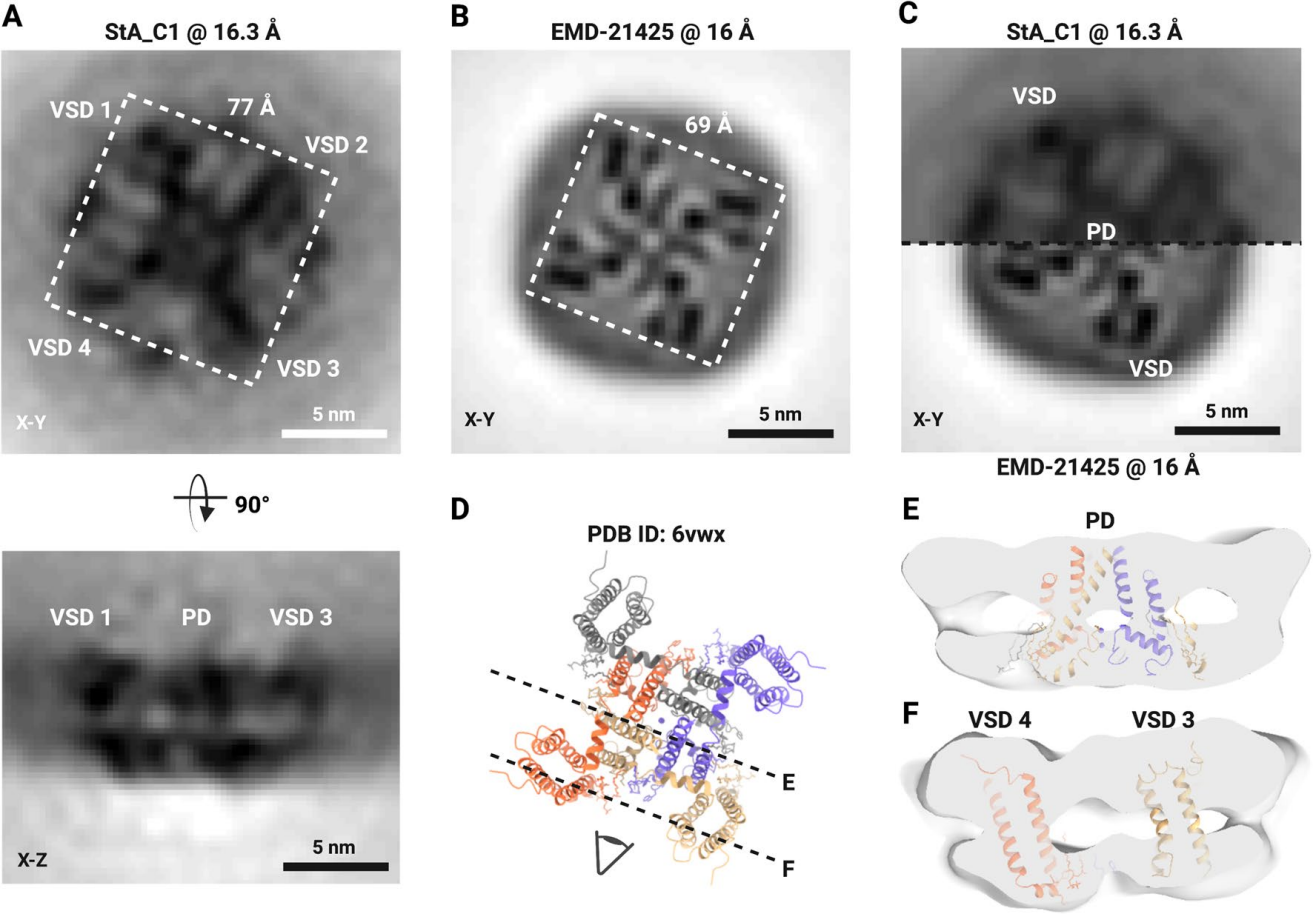
Structure of NaChBac in proteoliposomes and comparison to the structure in nanodiscs (EMD-21425). (**A**) StA map of NaChBac embedded in a liposome. (**B**) Cryo-EM map of NaChBac embedded in nanodisc. (**C**) StA map (above) at 16.3 Å compared with the recalculated cryo-EM structure (EMD-21425) at 16 Å resolution (below). The volumes were mutually aligned. The voltage sensing domain (VSD) and pore domain (PD) could be visualized as a tetrameric feature from the top of the StA structure. (**D**) The atomic model of EMD-21425 in an inactivated state. ((**E**) and (**F**) A segmented StA map of NaChBac embedded in a liposome overlaid with the atomic model built based on the single particle cryo-EM structure (EMD-21425). The model showed the density of the PD surrounded by four VSDs based on the fit of the PDB ID: 6vwx to the StA map.

We next performed StA. First, we used Dynamo to align all the subtomograms to a reference produced by manually aligning a small subset of particles and allowing the particles to rotate 360 degrees around the unit sphere (first two Euler angles) and applying high rotational symmetry around the vertical axis (not searching the last Euler angle). This resulted in a curved membrane with a defined density at the concave side of the membrane (Fig. 3). The membrane was less ordered at the edges of the box reflecting various sizes of proteoliposomes leading to "averaging out" of the bilayer. We next performed a classification of all the particles into 10 classes allowing the particles to rotate around the third Euler angle with limited rotations around the first two. This resulted in several classes resembling tetrameric features containing ˜42,100 particles, and the dataset was further cleaned to ˜31,800 particles with more classifications. Further classification based on rejecting the classes with poorly resolved membranes narrowed down the dataset to ˜25,500 particles. This particle set showed a tetrameric arrangement of density in the transmembrane domain (SI Appendix, Fig. S3). We took this particle set for further classification and refinement in RELION-4.0^41^. At this point there were still significant differences between the membrane curvatures of classes. We performed several rounds of classification and tried to auto-refine the resulting datasets with more or fewer particles, and ultimately, a dataset of 3,116 particles gave us the structure with a resolution of 16.3 Å (Fig. 4A and Fig. S5). During the outlined process we did not apply symmetry and only selected tetrameric-looking classes for further processing. The overview of the classification process is given in Fig. 3 and Fig. S4.

While the structure looked overall tetrameric, the symmetry was not perfect and refinement of the structure with C4 symmetry did not result in a better structure. Symmetry expansion also did not help to improve the structure. Comparing the structure to the intermediate class averages (Fig. 3), which we could not autorefine, we observed a heterogeneity in the positioning of the voltage-sensing domains. Finally, while we attempted multiple masks for the alignment, the best final alignment was achieved when the mask did not accommodate the densities of GFP. This could mean that the densities of GFP on a 15-long residue linker, while helpful for the localization of the proteins of interest, were flexible and did not help align the particles to the average.

The StA map clearly showed the domains of NaChBac in the lipid bilayer. At this domain-level resolution we could identify the tetrameric-appearing pore domain (PD) which contains eight full transmembrane helices, and the four smaller voltage-sensing domains (VSDs), each of which contains four transmembrane helices. Comparison of the StA reconstruction with the map from the single particle cryo-EM structure in lipid nanodisc reported previously (EMD-21425), resampled and low-pass filtered to 16 Å, showed overall similarity (Fig. 4B and C). The corresponding atomic model fitted the StA density well, considering the moderate resolution (Fig. 4D–F). Interestingly, the StA structure was ˜8 Å, or ˜10% wider compared to the structure in nanodiscs filtered to the same resolution (Fig. 4A,B).

## Discussion

We showed that in principle it is possible to identify and align small and mostly transmembrane proteins using StA and to obtain a correct structure, although at a modest resolution. Here, we could use the fused GFP in order to locate the proteins of interest in tomograms and to perform a rough alignment. However, for the final steps of the alignment the GFP was not useful, due to the flexibility of the linker region. Perhaps a more ordered interacting partner such as a specific toxin^42^, a Fab fragment^43^ or a legobody^44^ would be more useful to support particle alignment to the average. We did not use toxins or Fab fragments as those structures would be likely similar to the previously reported structures of purified channels^17,42^. The tetrameric NaChBac with a molecular weight of ˜120 kDa with mostly transmembrane residues is probably on the smaller side of the molecular weight that could be approached using the current technology. For our structural analysis we imaged the channels in thin ice, picked a large number of particles and used a combination of classification algorithms (Dynamo and RELION-4.0). Even with such optimizations, we could only obtain a resolution of ˜16 Å. While it is higher than the average resolution of StA structures deposited to the Electron Microscopy Data Bank in 2022 (20 Å), it only provides domain-level resolution. In our structural analysis we benefited from the defined tetrameric shape of the channel. We believe that structural analysis of even smaller proteins in membranes without large soluble domains or well defined overall shape such as monomeric GPCRs without interaction partners may be too ambitious at the current level of technology.

At the obtained resolution we could reliably observe the pore and the voltage-sensing domains of the channel. Thus, we could estimate that the structure of the channel in proteoliposomes is ˜10% wider than the structure reported in nanodiscs (EMD-21425) filtered to the same resolution. This could be physiologically relevant for two reasons. Firstly, nanodiscs were shown to confine the lipids inside the polymer^45^ and to modify the conformations of pentameric ligand-gated ion channels^46^. Secondly, molecular dynamics simulations for the K_v_ channel showed substantial motion of the VSDs^47^ that on average could make the proteins wider compared to the compact versions that are typically observed in nanodiscs. The possible motion of the VSD domains is another factor that potentially limits the obtainable resolution and could partially explain that the application of the C4 symmetry in our case did not lead to a much improved structure. While the observed increased protein diameter might be a result of the applied membrane potential, a recent report by Mandala and MacKinnon on the movements of the VSDs the Eag K_v_ channel showed that the cryo-EM structures of the channel with and without the transmembrane potential were overall similar in size. Therefore it is possible that in the case of NaChBac, the increased size of the channel is not a consequence of the applied potential but a result of imaging of the channel in a lipid bilayer without physical restraints.

With future improvements in detectors^48–50^ and phase plates^51^ it may be possible to get higher quality data and consequently higher resolution of NaChBac or proteins of similar sizes in proteoliposomes. Further sample optimization to unify the size of proteoliposomes would reduce the structural heterogeneity and could lead to improved resolution. Higher number of particles could improve the resolution; they may be obtained by processing more tomograms and picking more protein copies for StA. While the reconstruction of tomograms can be streamlined^35,52^, we did not manage to automate particle picking and had to pick them manually which is a very laborious process. Developments in machine learning and the potential applications for particle picking^53–55^ could streamline particle picking in the future. Our dataset will be on the difficult side for such applications. Obtaining higher resolution structures will enable the understanding of the factors influencing the conformations of sodium channels and their regulation by biophysical stimuli.

## Materials and methods

### Expression and purification of NaChBac

The cDNA for full-length NaChBac was cloned into pET21a with an amino terminal His_6_-GFP^A206K^. Overexpression in *E. coli* C41(DE3) cells was induced with 0.25 mM IPTG (final concentration) at 22 °C when the OD_600_ reached 0.8 to 1.0. Cells were harvested after 20-h incubation at 22 °C, and cell pellets were resuspended in a buffer containing 25 mM Tris, pH 8.5, and 300 mM NaCl. Cells were disrupted by high pressure homogenization, and insoluble fractions were removed by centrifugation at 30,000 × *g* for 30 min. The supernatant was subjected to ultracentrifugation at 150,000 × *g* for 2 h. The membrane-containing pellets were resuspended in extraction buffer containing 25 mM Tris, pH 8.5, 300 mM NaCl, 20 mM imidazole, and 1% (wt/vol) DDM, incubated at 4 °C for 2 h, and subsequently centrifuged at 30,000 × *g* for 30 min. The supernatant was applied to Ni–NTA resin, washed with 20 column volumes of wash buffer containing 25 mM Tris, pH 8.5, 300 mM NaCl, 20–80 mM imidazole, and 0.1% DDM. Target proteins were eluted with 3 column volumes of elution buffer containing 25 mM Tris, pH 8.5, 300 mM NaCl, 250 mM imidazole, and 0.1% DDM. After concentration, proteins were further purified by SEC (Superose 6 Increase 10/300 GL; GE Healthcare) in a running buffer containing 25 mM Tris, pH 8.5, 150 mM KCl, and 0.1% DDM.

### NaChBac reconstitution into liposomes

The protocol for proteoliposome reconstitution was carried out as described before^56^, with minor modifications. *E. coli* Polar Lipid Extract (Avanti Polar Lipids) dissolved in 400 μL chloroform at 25 mg/mL was dried to a thin film under a gentle stream of nitrogen and resuspended in 1 mL reconstitution buffer containing 25 mM HEPES, pH 7.0, and 150 mM KCl. After water-bath sonication for 5 min, the lipid solution was frozen with liquid nitrogen and thawed in water bath for 10 times, and the lipid solution was subject to repeated extrusion through 100 nm filters. To destabilize the liposomes prior to protein reconstitution, they were incubated with 2% (wt/vol) DDM (Anatrace) as a final concentration at 25 °C for 2 h. Subsequently, purified NaChBac was added at a lipid-to-protein ratio of 2:1 (wt/wt). After incubation at 4 °C for 1 h, the lipid-protein-detergent mixture was loaded into a dialysis bag, and dialyzed against the reconstitution buffer at 4 °C for approximately one week with gentle rotation. Following dialysis, and Bio-Beads SM-2 resin (Bio-Rad) 0.4 g was added to the sample to remove residual detergents from the proteoliposomes. After incubation at 4 °C overnight, Bio-beads were removed through filtration, and the proteoliposomes were ready for liposome flux assays.

### Density gradient centrifugation

Sucrose gradients were prepared in SW 40 Ti ultracentrifuge tubes (Beckman Coulter) on a Biocomp Gradient Master (ScienceServices, Munich) based on the method of Coombs and Watts^57^. Concentrated sucrose solution (25 mM HEPES, pH 7.0, 150 mM KCl, and 1 M sucrose) was layered under an equal volume of light solution (25 mM HEPES, pH 7.0, 150 mM KCl, and 0.3 M sucrose) in centrifuge tubes. The tubes were closed with caps to expel all air, and the gradient (10–30%) was formed by rotation. A 400 μL volume was removed from the top of each tube before the sample was added: 400 μL liposome sample with or without protein was loaded per tube and centrifuged at 130,000 × g for 18 h at 4 °C. After ultracentrifugation, opaque liposome bands were collected with a syringe and diluted with reconstitution buffer to remove most of the sucrose. Liposomes were pelleted at 90,000 × *g* for 1 h at 4 °C and resuspended in the reconstitution buffer, and samples were identified by SDS-PAGE.

### Negative-staining EM

Proteoliposome samples were diluted in a buffer containing 25 mM HEPES, pH 7.0, and 150 mM KCl to a series concentration. The sample was adsorbed to freshly glow-discharged carbon-coated grids, rinsed with several drops of the dilution buffer, and stained with 1% uranyl acetate. Images were recorded at a magnification of 49,000 × with defocus values ranging from − 2.5 to − 3.5 µm, resulting in a pixel size of 2.26 Å/pixel (Spirit Biotwin, FEI). As most of the protein was inserted with GFP located in the liposome lumen it was not possible to screen protein insertion with negative staining EM.

### Proteoliposome flux assay

The proteoliposomes prepared in 150 mM KCl were diluted 100-fold into 3 mM KCl solution (25 mM HEPES, pH 7.0, and 3 mM KCl) containing 1 μM of JC-1 (5P,5P,6,6P-tetrachloro-1,1,3,3P-tetraethylbenzimadazolylcarbocyanine iodide, Invitrogen). After the JC-1 fluorescence stabilized, valinomycin was added into the system to a final concentration of 1 μM. The fluorescence signal of the J-aggregates (λ_ex_ = 480 nm, λ_em_ = 595 nm) was monitored upon addition of valinomycin, whichinitiated K^+^ efflux from all the proteoliposomes until the external K^+^ concentration was increased by the addition of 2.5 M KCl solution. The normalized data were averaged across three independent measurements and the mean and standard errors are reported.

### Preparation of proteoliposomes with polarized membranes for Cryo-EM

The proteoliposomes prepared above were diluted into 10-nm gold fiducial markers in 3 mM KCl solution (25 mM HEPES, pH 7.0, and 3 mM KCl), and 1 μM valinomycin was added followed by a 5 min incubations on ice. An aliquot (2 μL) of this polarized proteoliposome solution was applied onto a glow-discharged holey gold grid (Quantifoil Au R2/2, 400 mesh). After incubating the sample on the grid for 3 min at 10 °C with a humidity of 100%, the grid was manually blotted from the side using a filter paper. Another 2 μL of the polarized proteoliposome solution was applied to the same grid for 15 s, and then the grid was blotted with Whatman^®^ No. 1 filter paper and plunge-frozen in liquid ethane (Vitrobot Mark IV, Thermo Fisher Scientific).

### Cryo-ET data collection, image processing, and subtomogram averaging

Imaging was performed on a Titan Krios G2 Cryo-TEM (Thermo Fisher Scientific) with a K3 direct detection camera (Gatan) and a BioQuantum imaging filter slit width of 20 eV (Gatan) operated by SerialEM software^58^. Tomographic series were acquired using a dose-symmetric scheme^59^, with tilt range ± 45°, 3° angular increment and defoci between − 2.5 and − 3.5 μm. The acquisition magnification was 81,000 × , resulting in a calibrated pixel size of 1.39 Å. The electron dose for every untilted image was increased to around 20 e^−^/Å^2^, and tilt images were recorded as 10-frame movies in counting mode and a total dose per tilt series of around 130 e^−^/Å^2^.

Data processing was performed using early versions of tomoBEAR^35^ implemented as a set of Matlab scripts. Frames were aligned and motion-corrected using MotionCor2^36^. Tilt series were aligned using 10-nm gold fiducial markers by IMOD^39,52^. Contrast transfer function (CTF) estimation was performed using defocus values measured by Gctf^37^ for each projection. A total of 98 tomograms and the four binned reconstructions were generated from CTF-corrected, aligned stacks using weighted back projection in IMOD. Attempts to identify particles with the use template matching with EMD-21425 as a template on binned 8 tomograms with and without nonlinear anisotropic diffusion were unsuccessful.We further attempted to parameterise the membranes and perform subtomogram classification in order to separate the particles from empty membranes. This approach also did not work due to a low fraction of proteins on a membrane, small size of the protein and a significant heterogeneity in the curvature of proteoliposomes.

Subtomogram positions (~ 86,000) were generously picked manually from 4-times binned tomograms and extracted with a box size of 128 cubic voxels from 2-times binned tomograms using the Dynamo Catalogue system^40^. Initial alignment was done manually on ~ 200 particles, and the center of four GFPs and the direction of the central axis based on the membrane were defined using *dynamo_gallery* after which the roughly aligned particles were summed up low-pass-filtered to 40 Å. This volume was used as an initial reference for the global alignment of all subtomograms, resulting in all particles aligned to the same Z height in 3D. Several rounds of initial classification by multireference alignment were used to remove junk particles, with the first step 360° in-plane search (XY plane) being performed on all particles in C1, and a large soft-edged sphere mask was applied for the particle cleaning. Further multireference alignment and averaging with a soft-edged ellipsoid alignment mask was applied throughout and the averaging results showed a prominent tetrameric feature in between the lipid bilayer in C1. In total, ~ 25,500 particles from good class averages were then subjected to the RELION-4.0^41^ for further 3D classification and refinement. Final converged averages were formed by 3116 particles in C1 at 16.3 Å resolution, and a smaller sphere mask was applied to improve the resolution. No symmetry was applied during processing.

The panels for the Fig. 1a were created with BioRender.com.

## Supplementary Information

Supplementary Information.

## Data Availability

The structure was deposited to the EMDB with an accession code EMD-17163. The original data were deposited to EMPIAR with an accession code EMPIAR-11576.
