# Supplementary material for: Determining the structure of the bacterial voltage-gated sodium channel NaChBac embedded in liposomes by cryo electron tomography and subtomogram averaging
Source: Sci Rep. 2023 Jul 17;13:11523. doi: 10.1038/s41598-023-38027-7 (PMC10352297; doi:10.1038/s41598-023-38027-7)
Supplement: Supplementary file 1 — Supplementary Information. [file 41598_2023_38027_MOESM1_ESM.pdf]

Supplementary Material

Determining The Structure of the Bacterial Voltage-gated Sodium Channel NaChBac Embedded in Liposomes by Cryo Electron Tomography and Subtomogram Averaging

Shih-Ying Scott Chang[1,2,3], Patricia M. Dijkman[1,2], Simon A. Wiessing[1], Misha Kudryashev[1,2,3,4]*

1. Max Planck Institute of Biophysics, Frankfurt on Main, Germany.

2. Buchmann Institute for Molecular Life Sciences, Goethe University of Frankfurt on Main, Germany.

3. *In Situ* Structural Biology, Max Delbrück Centre for Molecular Medicine in the Helmholtz Association, Berlin, Germany.

4. Institute of Medical Physics and Biophysics, Charité-Universitätsmedizin Berlin, Germany.

Email: mikhail.kudryashev@mdc-berlin.de

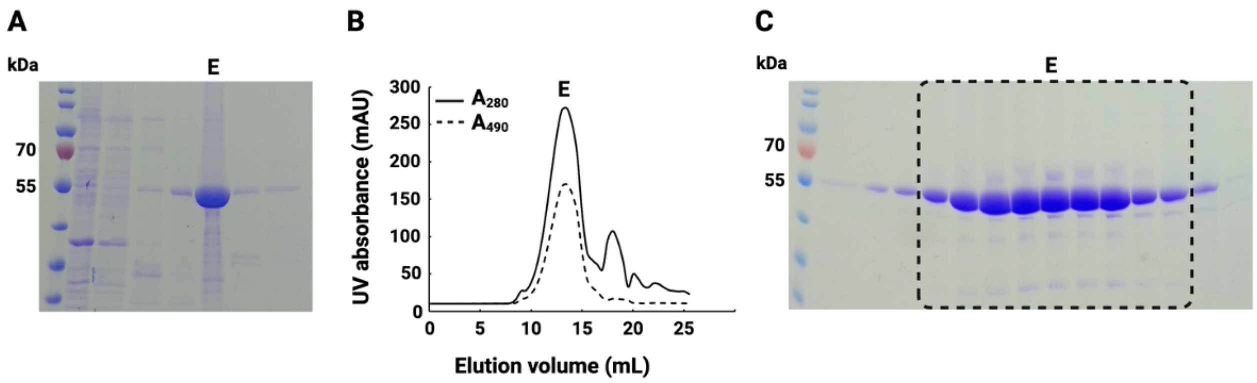

Figure S1 Purification of NaChBac. *(A) SDS-PAGE of protein purification with Ni-NTA column. E, elution. (B and C) SEC purification of NaChBac in DDM detergent with a monodispersed peak. E, elution.*

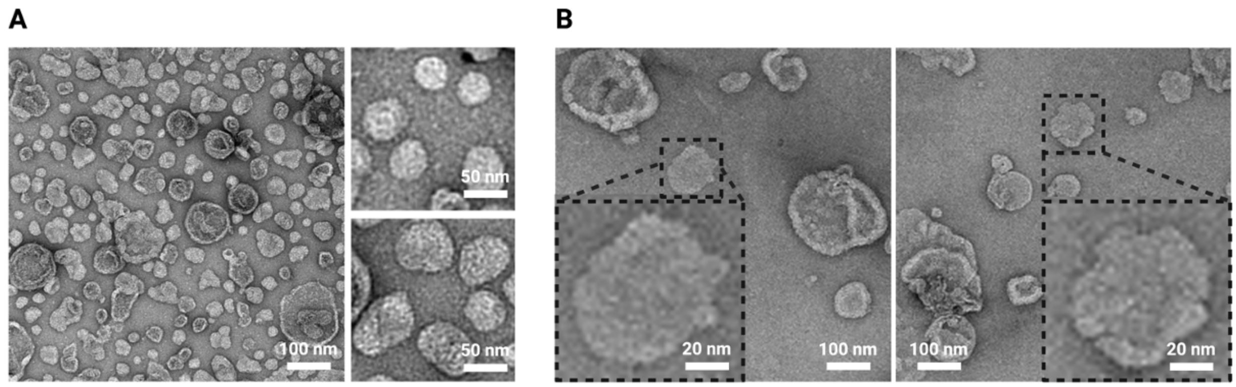

Figure S2 Negative-staining images of proteoliposomes. *(A) A negative-staining image of proteoliposomes with detergent removal entirely. The insets showed proteoliposomes with "sharp edge" features. (B) Negative-staining images of proteoliposomes with residual detergent during dialysis. The insets showed proteoliposomes with "fluid edge" features.*

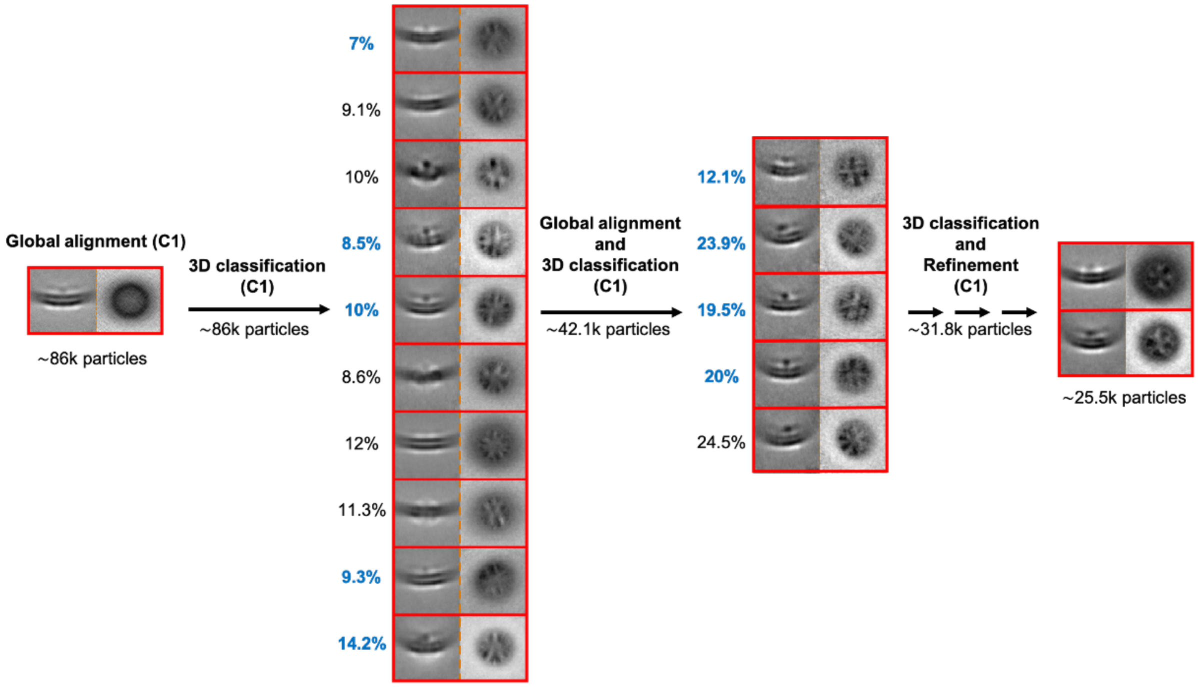

Figure S3 Strategy for the dataset cleaning by classification with Dynamo, without symmetry. *The images show central slices through the averages in XZ and XY directions, produced by Dynamo (D. Castano-Diez et al, J. Struct. Biol, 2012, 178:139-151). First, ˜86,000 particles were aligned globally and were implemented to 3D classification for the particle cleaning. ˜42,100 particles were selected to perform further classification. After collecting good particles (˜31,800) from previous steps, multiple rounds of 3D classification and refinement were performed, and ˜25,500 particles were then subjected to the RELION-4.0 for further 3D classification and refinement.*

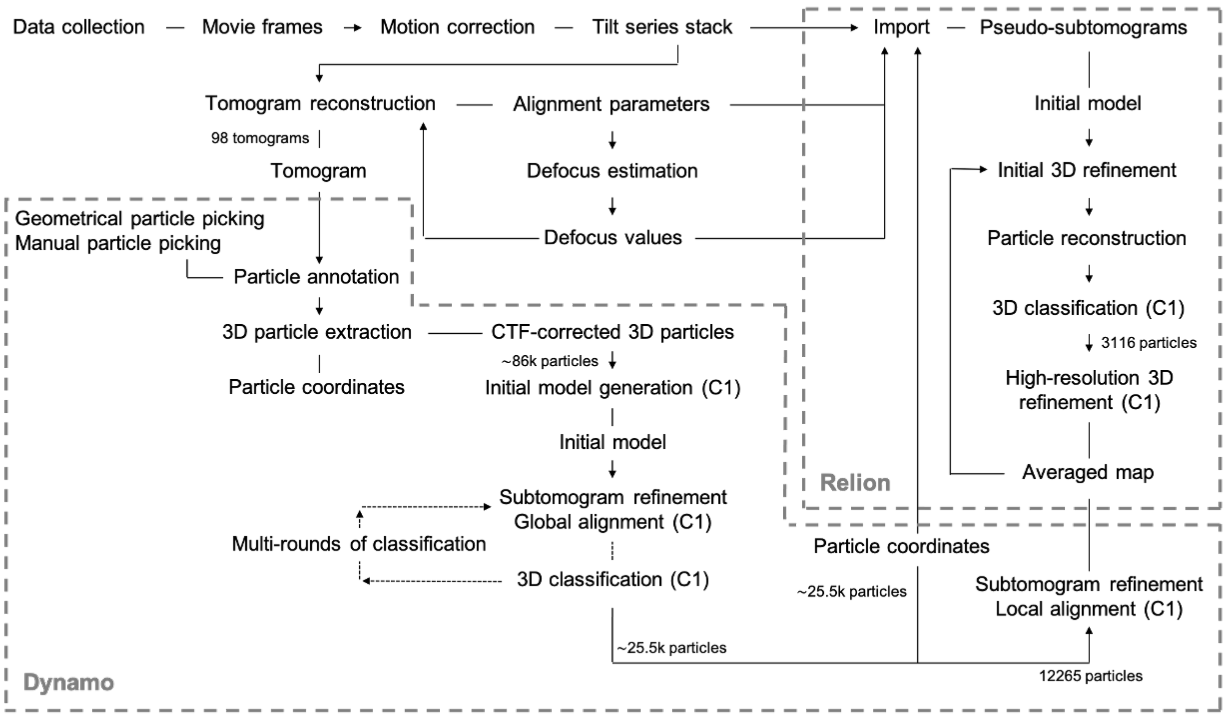

Figure S4 Workflow for tomogram reconstruction and subtomogram averaging. *Tilt series stack alignment, tomographic reconstruction, particle annotation, 3D classification, and subtomogram averaging were shown in the workflow.*

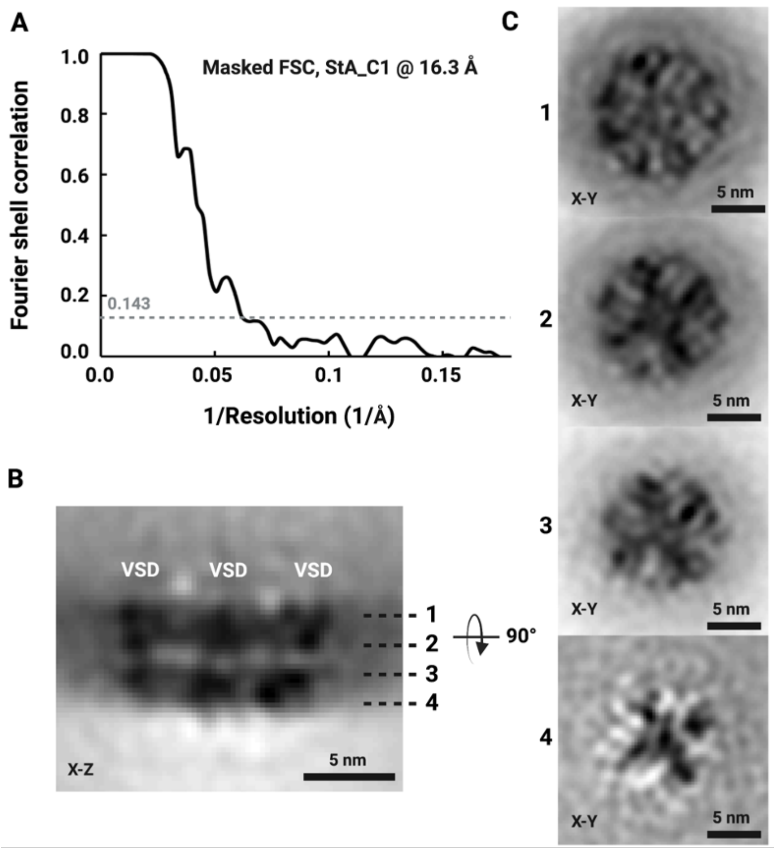

Figure S5 FSC curve of the masked map and slices through the structure of NaChBac map in liposomes along the z-axis. *(A) Gold standard FSC (criteria 0.143) curve of the masked map with a reported resolution of 16.3 Å for the map with C1 symmetry. (B and C) The voltage sensing domain (VSD) and pore domain (PD) could be visualized as a tetrameric feature in the X-Y plane along the z-axis.*

Table S1 Data Processing Statistics

	NaChBac in liposomes; EMD-17163
**Data collection and processing**	
Microscope	Titan Krios G2
Magnification	81,000 x
Voltage (kV), Cs	300 kV, 2.7 mm
Total electron dose (e^-/A^2)	˜130
Defocus range (µm)	-2.5 to -3.5
Camera	Gatan K3 + BioQuantum image filter
Pixel size (Å)	1.393
Number of tomograms	98
Symmetry imposed	C1
Initial particles	˜86,000
Final particles	3,116
Refinement method	Independent half-set
Map resolution (Å)	16.3
FSC threshold	0.143
